# Contact allergens in moisturizers in preventative emollient therapy – A systematic review

**DOI:** 10.1002/clt2.12150

**Published:** 2022-06-05

**Authors:** Klaudia Ryczaj, Karolina Dumycz, Radoslaw Spiewak, Wojciech Feleszko

**Affiliations:** ^1^ Department of Pediatric Pneumonology and Allergy Medical University of Warsaw Warsaw Poland; ^2^ Doctoral School Medical University of Warsaw Warsaw Poland; ^3^ Department of Experimental Dermatology and Cosmetology Jagiellonian University Medical College Krakow Poland

**Keywords:** dermatology, food allergy, pediatrics

## Abstract

**Background:**

Results of preventative emollient therapy on atopic dermatitis and food allergy trials are inconsistent. In addition to the ingredients considered beneficial, the moisturizers may contain potentially harmful haptens. This study aimed to assess the prevalence of haptens in moisturizers used in studies to prevent atopic dermatitis or food allergy and assess their correlations to the trial results.

**Methods:**

A systematic search of studies investigating the role of emollient usage in preventing atopic dermatitis or food allergy in infants was performed from inception to December 2020. Haptens were identified based on the nine common patch test series (European, American, and Australian).

**Results:**

12 clinical trial studies were included in the review. In total, 16 different emollients were applied as an intervention. The vast majority (75%) of preparations contained at least one hapten from which several substances pose high allergic or irritant potential. Quantitative data synthesis of the findings regarding food allergy and atopic dermatitis prevention was not possible due to the significant heterogeneity of preparations used.

**Conclusions:**

Careful selection of emollient should consider the absence of potentially harmful ingredients, particularly when used in youngest children. Chronic skin exposure to haptens promotes the development of allergic contact dermatitis and moreover, via deterioration of the skin barrier and subclinical inflammation, may facilitate epicutaneous sensitization and promote atopic dermatitis; however further research is needed to validate our suppositions.

## To the Editor,

Despite promising results of preliminary preventative emollient therapy studies, more extensive research did not confirm the beneficial effect and hinted at relevant risks. Next to beneficial ingredients, the moisturizers may contain molecules with a sensitizing or irritating potential that may disrupt skin barrier and cause inflammation. A higher potential to absorb external substances due to thinner stratum corneum, immature barrier function, and a higher ratio of skin's surface area to body weight makes children more susceptible to harmful effects of chemicals in moisturizers.

This study aimed to assess the prevalence of contact allergens in moisturizers used in studies to prevent atopic dermatitis (AD) or food allergy and assess their correlations to the trial results.

For this paper, the term “contact allergens” is used synonymously to haptens to refer to small molecules capable of inducing allergic contact dermatitis (ACD).

A systematic search of studies investigating the role of emollient usage in preventing atopic dermatitis or food allergy in infants was performed from inception to December 2020 (PROSPERO ID: CRD42021233262).

Twelve clinical trials were included in the review. In each clinical trial, from one to five emollients were used to enhance the skin barrier, of which at least one contained contact allergens (Table 1). In total, 16 different preparations were applied as an intervention, including two bath oils. In 12 out of 16 preparations (75%), at least one hapten from the common patch test series was identified. The majority of the haptens were emollients, preservatives, and emulsifiers. More than half of identified contact allergens also possess irritating potential. Propylene glycol, identified in two emollients used in three clinical trials, is a well‐known skin irritant. Therefore, its usage in children under two is not recommended.^1^ Lanolin, despite the evident moisturizing quality, displays a considerable risk of development of ACD and is also not recommended to be used in children before the age of two.^1^ Propylene glycol and lanolin were used in 4 out of 12 clinical trials (33%). Phenoxyethanol, present in cosmetics used in six clinical trials, is a preservative with low sensitizing properties. However, due to its widespread use in cosmetics, sensitization rates are similar to lanolin. Another identified hapten, isopropyl myristate, is also used as a penetration enhancer in topical and transdermal formulations increases skin permeation by fitting into the lipid lamellae and changing the microstructure of the stratum corneum.^2^

**TABLE 1:** Characteristics of included trials and used cosmetics as an intervention

Study ID, Country, study design, DOI	Study design	OCEBM levels of evidence	Emollient intervention	Haptens present in preparation	Ceramide‐based emollient or 'emollient plus'	Duration, frequency and location of the intervention	Population (n)	Results of the study	AD and FA diagnostic criteria, limitations
Chalmers et al., (BEEP study) *UK, 2020* DOI: 10.1016/S0140‐6736(19)32984‐8	RCT	Level 2	Diprobase cream	Cetearyl alcohol	no	12 months ≥1x daily to the whole body (excluding scalp)	High‐risk of atopy birth cohort (*n* = 1394)	‐ no differences in eczema prevalence in intervention versus control group at 2 years of age ‐ higher incidence of food allergy in emollient group (7%) versus control group (5%) but without statistical significance ‐ allergic sensitization to a food allergen at age 1–3 years was similar in intervention and control groups ‐ parent report of immediate reaction to food allergen was increased in the intervention group ‐ increased risk of skin infections in intervention group (mean number of skin infections per child in 1 year was 0·23 (SD 0·68) in the emollient group versus 0·15 (0·46) in the control group)	‐ diagnosis of AD: the UKWP criteria ‐ diagnosis of FA: parental report, allergic sensitization, food challenge ‐ if required
			DoubleBase gel	Isopropyl myristate, Phenoxyethanol	no				
Skjerven et al., (PreventADALL study) *Norway, Sweden, 2020* DOI: 10.1016/S0140‐6736(19)32983‐6	RCT	Level 2	Paraffin bath oil^a^	none	no	8 months ≥5 days/week Bath in paraffin bath oil and facial cream	Standard‐risk newborns (*n* = 2397)	‐ no differences in eczema prevalence in any intervention groups versus control groups in ITT analysis ‐ in analyses for which missing outcome was addressed by multiple imputation, the risk of atopic dermatitis was significantly increased in the skin intervention group with a risk difference of 5·9% (2·0–9·7)	‐ diagnosis of AD: the UKWP and Hanifin and Rajka criteria
			Ceridal creme^b^	none	no				
Thitthiwong et al., *Thailand, 2020* DOI: 10.33192/Smj.2020.06	RCT	Level 2	Cream called “Cold cream” prepared by the hospital in which trial was conducted^a^	Stearyl alcohol, Propylene glycol	no	9 months ≥1x daily to the whole body excluding perioral and periorbital areas	High‐risk infants (*n* = 53)	‐ 52 infants finished the study ‐ none of the infants from intervention group developed AD, while in control group 4 children developed AD (*p* = 0.045)	‐ diagnosis of AD: based on the atopic dermatitis guidelines by Eichenfield et al., in 2014.
Yonezawa et al., *Japan, 2019* ^*S4,S5*^ DOI: 10.1111/1346–8138.14080 DOI: 10.1186/s13223‐019‐0385‐7	RCT	Level 2	Atopita Milky Lotion^b^	Lanolin, Sorbitan sesquioleate, Phenoxyethanol	no	3 months 1‐2x daily	Standard risk infants (*n* = 227)	‐ 155 participants were included in final analysis ‐no differences in prevalence of FA and AD at the age of 2 years were reported between intervention and control groups in intention to treat analysis and per protocol analysis ‐ different skin problems occurring during first 3 month of life were reported to be a risk factor for FA development (*p* = 0.015, *p* = 0.032) ‐ statically significant lower TEWL in the intervention group measured on face at the age of 3 months (mean ± SD, 14.69 ± 7.38 vs. 17.08 ± 8.26 g/m2 per h, *p* = 0.033)	‐ diagnosis of AD: self‐reported questionnaires administered to the parents (“many skin problems and using steroids” and “diagnosed atopic dermatitis” were both considered AD/eczema) ‐ diagnosis of FA: self‐reported food allergies
			Pigeon Baby Milk Lotion^b^	Ethylhexyl glycerin, Tocopherol, Stearyl Alcohol, Phenoxyethanol	Ceramide based				
Dissanayake et al., *Japan, 2019* DOI: 10.1159/000501636	RCT	Level 2	Locobase REPAIR Cream^b^	Sorbitan oleate	Ceramide based	6 months 2‐3x daily especially to cheeks and perioral areas, application to other parts of the body was allowed but not advised for or against	Standard‐risk newborns (*n* = 549) Skin care and synbiotics. (*n* = 137) Synbiotics only (*n* = 137) Skin care only (*n* = 138) No intervention (*n* = 137)	‐ no statistically significant differences in eczema and food allergy prevalence in received emollients group and no emollient group at 12 months of age ‐ no differences in EASI scores, TARC levels and sensitization rate (sIgE) in 9 months of age among children diagnosed with AD	‐ diagnosis of AD: the UKWP criteria, cumulative incidence of AD characterized as itchy skin condition lasting 2 or more months ‐ diagnosis of FA: self‐reported food allergies
McClanahan et al., *USA, 2019* DOI: 10.1111/jdv.15786	RCT	Level 2	Cetaphil Restoraderm^a^	Cetyl alcohol, Cetearyl alcohol, Panthenol, Tocopheryl acetate	Ceramide based and emollient plus	12 months, daily application to whole body excluding scalp and diaper area	High‐risk population (*n* = 100)	‐ fewer AD cases in intervention group were reported but without statistical significance ‐ there were more cases of reported contact dermatitis in the intervention versus control arms, 9.3% versus 4.3%, respectively, however, these events were not related to the study emollient ‐ no differences in TEWL, skin capacitance and skin pH at the age of 2, 6 and 12 months	‐ diagnosis of AD: the UKWP criteria adapted to identify incident cases of AD rather than a 12‐month period prevalence
Lowe et al., (PEBBLES study) *Austratia, 2018* DOI: 10.1111/bjd.15747	RCT	Level 2	Epiceram^b^	Phenoxyethanol, Sorbic acid	Ceramide based	6 months 2x daily Full skin surface	High‐risk infants (*n* = 80)	‐ visible trend for reduced AD and food sensitization prevalence in intervention group ‐ no differences in sensitization outcomes in ITT analysis ‐ significant reduction of food sensitization in PP analysis in the intervention group (*p* = 0.04) ‐ children who developed food sensitization in the intervention group had a later initiation of treatment ‐ no significant differences in TEWL, skin capacitance, pH and sebum concentration measurements in both groups	‐ diagnosis of AD: the UKWP criteria, observed by investigator
Kvenshagen et al., *Norway, 2014* DOI: 10.1016/j.aller.2014.06.003	nRCT	Level 3	Bath oil^b^	none	no	6 months 1x day Bath in bath oil and facial cream	Infants with xerosis up to 6 weeks of age (*n* = 56)	‐ infants from the intervention group had more often normal skin (*p* = 0.01) ‐ probable AD was observed in one child in intervention group and in six children in the control group (*p* = 0.1, nonsignificant)	‐ diagnosis of AD: term “probable AD” was defined as dry skin with observed scratching
			Ceridal Creme	none					
Horimukai et al., *Japan, 2014* DOI: 10.1016/j.jaci.2014.07.060	RCT	Level 2	2e (Douhet) emulsion^a^	Phenoxyethanol, Tocopherol	no	8 months ≥1x daily to whole body surface	High‐risk infants (*n* = 118)	‐ 32% fewer infants developed AD in intervention group by week 32 (*p* = 0.012), ‐ risk of AD was significantly lover in intervention group (hazard ratio, 0.48; 95% CI, 0.27–0.86) ‐ sensitization rate to egg white, evaluated by sIgE measurement, did not differ between intervention and control group ‐ the intervention group had significantly higher levels of stratum corneum hydration in the lower leg at weeks 12 and 24 compared to control group (*p* < 0.05)	‐ diagnosis AD: The diagnostic criteria for infantile eczema, AD, or both (AD/eczema) were developed based on a modification of the UKWP criteria
Simpson et al., *UK, USA, 2014* DOI: 10.1016/j.jaci.2014.08.005	RCT	Level 2	Aquaphor Healing Ointment	Lanolin, Panthenol	no	6 months 1x daily to whole body surface with exception of scalp	High‐risk infants (*n* = 124)	‐ daily emollient use significantly reduced the cumulative incidence of atopic dermatitis at 6 months (43% in the control group vs. 22% in the emollient group) with RR reduction of 50% (RR, 0.50; 95% CI, 0.28–0.9; *p* = 0.017)	‐ diagnosis AD: assessment by the dermatologist or dermatology specialist nurse
			Cetaphil Cream	Benzyl alcohol, Cetyl alcohol, Phenoxyethanol, Propylene glycol, Tocopheryl acetate	no				
			DoubleBase gel	Isopropyl myristate, Phenoxyethanol	no				
			Liquid paraffin^a^	none	no				
			Sunflower seed oil^a^	none	no				
Inoue et al., *Japan, 2013* DOI: 10.1684/ejd.2013.1960	nRCT	Level 3	DARDIA Lipo Cream (Intendis, Osaka, Japan)	Ethylhexyl glycerin	no	3 months, 2x daily	Standard risk newborns (*n* = 147)	‐ 104 of children completed the study ‐ AD prevalence at 4 months of age did not differ between intervention and control group (30/147 vs. 226/1405, *p* = 0.205) ‐ TEWL measurement tended to be higher in control group, but without statistical significance (median TEWL 16.15, IQR 13.00–21.90 vs. 17.55, IQR 13.68–24.30, *p* = 0.057)	‐ diagnosis AD: Hanifin and Rajka criteria ‐ as a control group data from other 4‐month‐old Japanese infants who were born in the same area was used
Simpson et al., *USA, 2010* DOI: 10.1016/j.jaad.2009.11.011	nRCT	Level 3	Cetaphil Cream	Benzyl alcohol, Cetyl alcohol, Phenoxyethanol, Propylene glycol, Tocopheryl acetate	no	90–773 days, 1x daily to whole body surface with exception of scalp and diaper area	High‐risk infants (*n* = 22)	‐ 3 out 20 (15%) infants developed AD by the day 547 from enrollment	‐ diagnosis AD: when all of the following were met: (1) the presence of eczema in typical locations, (2) pruritus, and (3) eczema that lasted for at least 2 weeks

^a^ composition given in the study.

^b^ composition obtained directly from the authors of the study; *AD*, atopic dermatitis; *UKWP*, The United Kingdom Working Party's*; FA*, food allergy; *TEWL*, Trans Epidermal Water Loss; *EASI*, Eczema Area and Severity Index; *TARC*, Thymus and activation‐regulated chemokine.

Manufacturers are not obligated to provide the amount and concentration of ingredients but only to show them in descending order. A Resolution on safety criteria for cosmetic products intended for infants, adopted by the Council of Europe (CM/ResAP[2012]1), indicates that toxic ingredients and potent allergens should not be present and that preservatives should be used at their lowest effective concentrations in these cosmetics. Unfortunately, due to the occurrence of more than one contact allergen in one cosmetic and their unknown concentrations, the risk of allergic sensitization to emollients in preventive AD therapy cannot be predicted.

Not every moisturizer improves skin barrier function; some even may impair skin barrier function by damaging the stratum corneum, removing skin lipids, increasing susceptibility to irritants, and accelerating trans‐epidermal water loss (TEWL).^3^ A “sensitive skin” animal model study showed that topical applications of several branded skin care products, marketed as “barrier repair” formulations, in fact negatively affected the skin barrier.^4^ Furthermore, prolonged use of emollients in preventive AD therapy may also lead to increased risk of skin infections by altering the microbiome and increasing the risk of AD and FA.

The only products in the trials that were free of common haptens were Ceridal Lipogel, paraffin‐based‐bath oils, liquid paraffin, and pure sunflower seed oil.

Studies are lacking in assessing the impact of haptens in skincare products on the epidermal barrier and inflammation. Mc Fadden et al.^5^ argued that prolonged skin exposure to haptens could cause a shift in Th cell phenotype toward Th2 and favor Th2 immune responses to third‐party antigens. That selective Th2 phenotype would favor the occurrence or exacerbations of AD and the development of IgE sensitization to proteins such as food or pollen.^6^ Such mechanism would provide a speed lane for sensitization to large molecular allergens in cosmetics, among which are food allergens like wheat hydrolysates. Moreover, it would explain the dose‐response relationship between emollient use and food and aeroallergen sensitization in the Enquiring About Tolerance (EAT) study population.^7^

Historically it was believed that ACD was rare in children due to limited exposure to haptens and immaturity of the immune system.^8^ Nowadays, it has become evident that ACD is among the most common pediatric skin disorders and may begin even in early infancy.^9^

There were limitations to our systematic review. Based on data available from published clinical trials, we could not assess a direct association between haptens contained in moisturizers and either AD or FA. Quantitative analysis was not possible due to the heterogeneity of extracted data; therefore, further research is needed to validate our suppositions.

We showed that the vast majority (75%) of emollients studied in clinical trials contain at least one common contact sensitizer. Of note, chronic skin exposure to sensitizing haptens increases the risk of ACD development and may facilitate epicutaneous sensitization and promote atopic dermatitis. Therefore, careful selection of emollients should avoid potentially harmful ingredients, including haptens, particularly when used in younger children.

## CONFLICT OF INTEREST

There are no conflicts of interest to declare. All authors have approved the manuscript and agreed to submission.

## ETHICS APPROVAL

Statement on ethics approval and consent – not applicable.

## AUTHOR CONTRIBUTIONS

**Klaudia Ryczaj:** Conceptualization (equal); Data curation (equal); Formal analysis (equal); Methodology (equal); Visualization (equal); Writing – original draft (equal). **Karolina Dumycz:** Data curation (equal); Formal analysis (equal); Methodology (equal); Visualization (equal); Writing – original draft (equal). **Radoslaw Spiewak:** Methodology (equal); Writing – review & editing (equal). **Wojciech Feleszko:** Conceptualization (equal); Methodology (equal); Supervision (equal); Writing – review & editing (equal).

## Data Availability

Data available on request from the authors.
